# An Aeromagnetic Compensation Strategy for Large UAVs

**DOI:** 10.3390/s24123775

**Published:** 2024-06-10

**Authors:** Liwei Ye, Zhentao Yu, Yaxun Zhang, Cheng Chi, Pu Cheng, Jie Chen

**Affiliations:** 1 Qingdao Innovation and Development Center, Harbin Engineering University, Qingdao 266500, China; zhangyaxun@hrbeu.edu.cn; 2 Institute of Remote Sensing, Navy Submarine Academy, Qingdao 266000, China; cheng.chihhu@163.com (C.C.); chengpu@nudt.edu.cn (P.C.); grass2009@163.com (J.C.)

**Keywords:** aeromagnetic compensation, unmanned aerial vehicles, multicollinearity, Lasso regularization Newton iteration (LRNM)

## Abstract

Aeromagnetic surveys are widely used in geological exploration, mineral resource assessment, environmental monitoring, military reconnaissance, and other areas. It is necessary to perform magnetic compensation for interference in these fields. In recent years, large unmanned aerial vehicles (UAVs) have been more suitable for magnetic detection missions because of the greater loads they can carry. This article proposes some methods for the magnetic compensation of large multiload UAVs. Because of the interference of the large platform and instrument noise, the standard deviations (stds) of the compensation data used in this paper are larger. At the beginning of this article, using the traditional T-L model, we avoid the shortcomings of the anti-magnetic interference ability of triaxial magnetic gate magnetometers. The direction cosine information is obtained by using an inertial navigation system, the global positioning system, and a triaxial magnetic gate magnetometer. Then, we increase the amplitude of the maneuvers in the compensation process; this reduces the multicollinearity problems in the compensation matrix to a certain extent, but it also results in greater magnetic field interference. Lastly, we employ the method of Lasso regularization Newton iteration (LRNM). Compared to the traditional methods of least squares (LS) and singular value decomposition (SVD), LRNM provides improvements of 34% and 27%, respectively. In summary, this series of schemes can be used to perform effective compensation for large multi-load UAVs and improve the actual use of large UAVs, making them more accurate in the measurement of aeromagnetic survey data.

## 1. Introduction

Aeromagnetic surveying is a geophysical method for measuring the intensity or gradient of a magnetic field using airborne instruments. The main purpose of an aeromagnetic survey is to study the underground geological geology and find mineral resources. The data of geomagnetic fields are collected using an aircraft magnetometer, with supporting equipment installed, for the scheduled measurement of a certain altitude. The data are then analyzed to map out the geomagnetic field and to infer the underground rock structure and the locations of the minerals [1,2,3,4,5]. In the course of the aeromagnetic process, the flight direction and changes in the motion posture will cause the magnetic elements of the UAV to produce a magnetic field, which interferes with the magnetometer and affects the accuracy of the measurement. In order to ensure that the magnetometer is able to accurately measure the geomagnetic field, magnetic compensation is required to offset this interference [6,7].

Compensation is important for the position of an aeromagnetic survey; the process of eliminating these interference fields is the magnetic compensation of the magnetic field [8,9,10]. In 1950, Tolles and Lawson proposed a model of magnetic field interference comprising the (1) permanent magnetic field, (2) induction field, and (3) eddy current field, and then established a compensation matrix to solve for the compensation coefficients [11,12,13]. This classic model is still in use today. Methods for solving the compensation matrix have also been a research hotspot in recent years. Zhao [14] proposed the recursive least squares method, Gu [15] proposed the singular value decomposition method, SU [16] proposed the ridge regression method, and Yu [17] proposed the method of using a residual neural network for compensation. For the selection of flight compensation platforms, Chen [18] proposed a compensation scheme for helicopter platforms, Qiao [19] proposed a magnetic compensation scheme for multi-rotor drones, and MU [20] proposed a series of compensation schemes for small drones as carriers. Regarding the multicollinearity issues of the T-L model, Zhao [21] and Ge [22] proposed methods for improving the model.

In the actual flight compensation process, there are many problems. First, the precision of the three-axial magnetic fluxgate magnetometer and its anti-noise interference ability are bad [23]. The interference that occurs in the process of the UAV turning inevitably influences the precision of the triaxial fluxgate. An increase in error is further transmitted to the solution of the compensation coefficient, resulting in low precision, so the use of a triaxial fluxgate alone to obtain the compensation matrix is not sufficient [24]. Secondly, the compensation matrix obtained during the compensation process has the problem of multicollinearity. If large maneuvering actions are used, this will reduce the problem of multicollinearity, but large maneuvering actions also make the interference field larger during the compensation process [25]. At present, in order to increase the detection efficiency, the flying UAV platform is often large in volume and equipped with a large load; combined with the superimposed effects of the large maneuvers described above, this leads to a larger interference field than those addressed in previous articles.

In this article, a model for improving the triaxial magnetic gate error is first introduced, using the attitude angle obtained from an inertial navigation system (INS) and the global positioning system (GPS) to build a new compensation matrix, which is then combined with the traditional T-L model. Then, a new compensation optimization algorithm, Lasso regularization Newton iteration (LRNM), is proposed in Section 2, which can also reduce the problem of multicollinearity to a certain extent. We present the experiment in Section 3. In Section 4, through a comparison of the traditional solution of the least squares method and the singular value decomposition method, it can be seen that the new optimization algorithm proposed in this paper achieves a better compensation effect. By combining these compensation strategies, this method can be used to compensate for the interference of large UAVs. Finally, the contents of this article and future trends are summarized in Section 5.

## 2. Principles and Methods

### 2.1. T-L Model

Before an aircraft can conduct an aeromagnetic survey, it is necessary to install a data instrument, an optical pump magnetometer (OPM), a triaxial magnetic vector fluxgate magnetometer, an inertial navigation system, and a global positioning system unit. Because these instruments affect magnetic detection, it is necessary to perform magnetic compensation.

**Figure 1:**
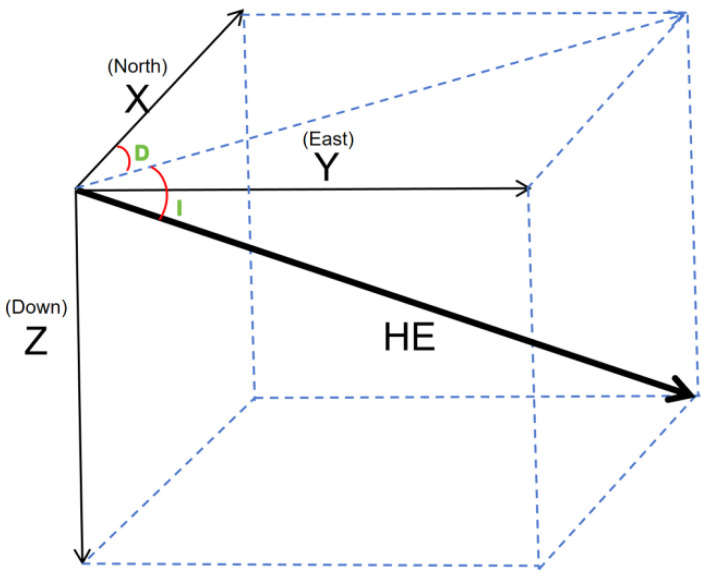
Coordinate system of the T-L model.

In 1950, Tolles and Lawson proposed a model [26] that was later called the Tolles–Lawson model. The total magnetic field of the optical pump magnetometer in actual operation is composed of the UAV’s interference field, the magnetic anomaly signal, and Earth’s general magnetic field.

(1) $$H_{all}=H_{d}+H_{y}+H_{e}$$

where $\;H_{d}$ represents the aircraft interference field, $\;H_{y}$ represents the magnetic anomaly signal, and $\;H_{e}\;$ represents the geomagnetic field. According to the conclusion of the T-L model, the aircraft interference field can be divided into three parts: the permanent magnetic field, the induction field, and the eddy current field. The formula is as follows:

(2) $$H_{d}=H_{p}+H_{i}+H_{r}$$

where ${\;H}_{p}$ represents the permanent magnetic field, ${\;H}_{i}$ represents the induction field, $and\;H_{r}$ represents the eddy current field. The coordinate system used in the T-L model is based on aircraft in air motion. The aircraft center is taken as the origin *O*, where the X axis is parallel to the fuselage, pointing in the direction of the aircraft’s movement, the Y axis is perpendicular to the fuselage, pointing in the direction of the wing, and the Z axis perpendicular to the UAV, pointing to the ground, as shown in Figure 1. $H_{e}\;$ denotes the geomagnetic field, α  is the angle of the X axis and the geomagnetic field, β  is the angle of the Y axis and the geomagnetic field, and γ  is the angle of the Z axis and the geomagnetic field, *D* is magnetic declination, *I* is magnetic dip. The calculation methods for the angles are as follows:

(3) $$cos\;\alpha =\frac{H_{1}}{\sqrt{H_{1}^{2}+H_{2}^{2}+H_{3}^{2}}}$$

(4) $$cos\;\beta =\frac{H_{2}}{\sqrt{H_{1}^{2}+H_{2}^{2}+H_{3}^{2}}}$$

(5) $$cos\;\gamma =\frac{H_{3}}{\sqrt{H_{1}^{2}+H_{2}^{2}+H_{3}^{2}}}$$

Here, $H_{1}$, $H_{2}$, and $H_{3}$ are the data obtained by the three axes of the vector fluxgate magnetometer. And the sum of the vectors of the three axes is equal to the value of Earth’s magnetic field.

(6) $$H_{e}=\sqrt{H_{1}^{2}+H_{2}^{2}+H_{3}^{2}}$$

(1) Regarding the permanent magnetic field, the aircraft will produce a fixed magnetic field in the course of its magnetic measurement. The magnetic fields will have an impact on the magnetometer on the UAV, however, the magnitude of this interference remains constant over time.
  (7) $$B_{p}=\left[\begin{array}{c} P_{1}\\ P_{2}\\ P_{3} \end{array}\right]$$
  where $B_{p}$ is the component of the three-axis of the UAV’s permanent magnetic field, which is given in vector form. The following is the $H_{p}$ formula:
  (8) $$H_{p}=\frac{H_{e}}{\left|H_{e}\right|}\cdot B_{p}=P_{1}cos\;\alpha +P_{2}cos\;\beta +P_{3}cos\;\gamma ={\left[\begin{array}{c} cos\;\alpha \\ cos\;\beta \\ cos\;\gamma \end{array}\right]}^{T}\cdot \left[\begin{array}{c} P_{1}\\ P_{2}\\ P_{3} \end{array}\right]$$
(2) The induction field in an aircraft is significantly influenced by various soft magnetic materials distributed throughout the structure, which vary in shape and are integral to the aircraft’s design. This field is closely linked to the UAV’s flight direction, motion posture, and velocity. Changes in the UAV’s motion modify the magnetic fields produced by the onboard magnetic elements aligned with the three axes. These changes magnetize the airborne soft magnetic materials, impacting the measurements recorded by the airborne magnetometer. This interference highlights the need to consider the aircraft’s design and operational parameters in aeromagnetic surveys to ensure accurate data collection.
  (9) $$B_{i}=\left[\begin{array}{ccc} I_{11} & I_{12} & I_{13}\\ I_{21} & I_{22} & I_{23}\\ I_{31} & I_{32} & I_{33} \end{array}\right]\cdot \left|H_{e}\right|\left[\begin{array}{c} cos\;\alpha \\ cos\;\beta \\ cos\;\gamma \end{array}\right]$$
  where $B_{i\;}$ represents the vector in the UAV’s induction field. $\;\;I_{11}$, ${\;I}_{12}$, ${\;and\;I}_{13}$ denote the components of Earth’s magnetic field on the Y axis and are the magnetization effect coefficients for soft magnetic materials on the X, Y, and Z axes of the aircraft coordinates. ${\;I}_{21}$, ${\;I}_{22}$, and ${\;I}_{23}$ indicate the components of Earth’s magnetic field on the Y axis and are the magnetization effect coefficients for soft magnetic materials on the X, Y, and Z axes of the aircraft coordinates. $\;I_{31}$, ${\;I}_{32}$, and ${\;I}_{33}$ denote the components of Earth’s magnetic field on the Y axis and are the magnetization effect coefficients for soft magnetic materials on the X, Y, and Z axes of the aircraft coordinates. The formula for $H_{i}$ is as follows:
  (10) $$H_{i}=\frac{H_{e}}{\left|H_{e}\right|}\cdot B_{i}={\left[\begin{array}{c} cos\;\alpha \\ cos\;\beta \\ cos\;\gamma \end{array}\right]}^{T}\cdot \left[\begin{array}{ccc} I_{11} & I_{12} & I_{13}\\ I_{21} & I_{22} & I_{23}\\ I_{31} & I_{32} & I_{33} \end{array}\right]\cdot \left|H_{e}\right|\left[\begin{array}{c} cos\;\alpha \\ cos\;\beta \\ cos\;\gamma \end{array}\right]$$
(3) The eddy current field is generated during aeromagnetic measurements when the movement of the aircraft induces currents in its conductive components (such as the aluminum alloy fuselage), producing an eddy current, which produces the eddy current field. This magnetic field is dynamic and related to the speed and acceleration of the UAV. Therefore, to maintain the precision of the magnetic field measurements, compensation for the aircraft’s inherent magnetic field is essential.
  (11) $$B_{r}=\left[\begin{array}{ccc} r_{11} & r_{12} & r_{13}\\ r_{21} & r_{22} & r_{23}\\ r_{31} & r_{32} & r_{33} \end{array}\right]\cdot \left|H_{e}\right|\left[\begin{array}{c} \frac{dcos\;\alpha }{dt}\\ \frac{dcos\;\beta }{dt}\\ \frac{dcos\;\gamma }{dt} \end{array}\right]$$
  $where\;B_{r}$ represents the vector of the eddy current field. $\frac{dcos\;\alpha }{dt}$, $\frac{dcos\;\beta }{dt}$, $\frac{dcos\;\gamma }{dt}$ are derivatives of the cosine angle. r is the eddy current field coefficient of the magnetic component of the X, Y, and Z axes. The formula for $H_{r}$ is as follows:
  (12) $$H_{r}=\frac{H_{e}}{\left|H_{e}\right|}\cdot B_{r}={\left[\begin{array}{c} cos\;\alpha \\ cos\;\beta \\ cos\;\gamma \end{array}\right]}^{T}\cdot \left[\begin{array}{ccc} r_{11} & r_{12} & r_{13}\\ r_{21} & r_{22} & r_{23}\\ r_{31} & r_{32} & r_{33} \end{array}\right]\cdot \left|H_{e}\right|\left[\begin{array}{c} \frac{dcos\;\alpha }{dt}\\ \frac{dcos\;\beta }{dt}\\ \frac{dcos\;\gamma }{dt} \end{array}\right]$$

According to the above derivation, the UAV’s interference field can be summarized as follows:

(13) $$H_{d}=H_{P}+H_{i}+H_{r}=\sum_{i=1}^{3}p_{i}c_{i}+H_{e}\sum_{i=1}^{3}\sum_{j=1}^{3}I_{ij}c_{i}c_{j}+H_{e}\sum_{i=1}^{3}\sum_{j=1}^{3}r_{ij}{\dot{c}}_{i}c_{j}\;i,i=1,2,3$$

Here, $c_{i\;}$ represents the direction cosines, cos α,  cos β, and cos γ. ${\dot{c}}_{i}$ represents the derivative of the direction cosines of the equation, cos α, cos β, and cos γ. $p_{i}$ represents the coefficient of the permanent field, $I_{ij}$ represents the coefficient of the induction field, and $r_{ij}$ represents the coefficient of the eddy current field.

(14) $$H_{d}=A\times C$$

Here, ${\;H}_{d}$ is still the interference field produced by the UAV, and A represents a vector, including [$a_{1}$, $a_{2}$, $a_{3}$, $a_{1}a_{1}$, $a_{1}a_{2}$, $a_{1}a_{3}$, $a_{2}a_{1}$, $a_{2}a_{2}$, $a_{2}a_{3}$, $a_{3}a_{1}$, $a_{3}a_{2}$, $a_{3}a_{3}$, ${\dot{a}}_{1}a_{1}$, ${\dot{a}}_{1}a_{2}$, ${\dot{a}}_{1}a_{3}$, ${\dot{a}}_{2}a_{1}$, ${\dot{a}}_{2}a_{2}$, ${\dot{a}}_{2}a_{3}$, ${\dot{a}}_{3}a_{1}$, ${\dot{a}}_{3}a_{2}$, ${\dot{a}}_{3}a_{3}$]. ${\dot{a}}_{i}$ represents the derivative of the three directions cosines of time. Since $a_{2}a_{1}$ and $a_{1}a_{2}$, ${\;a}_{2}a_{3}$ and $a_{3}a_{2}$, ${and\;a}_{1}a_{3}\;and\;{\;a}_{3}a_{1}$ have the same physical meanings, only one of each is kept. C represents the unknown solution coefficient of 18, C = [c1, c2, c3, …, c18].

We can see that the vector that is composed of the direction cosines, which can be measured by the fluxgate magnetometer. $H_{d}$ can be obtained by the pump sensor, so we can acquire C by solving the equation. C, in this case, is the compensation coefficient in the T-L equation.

$$A\;\mathrm{becomes}\;\mathrm{the}\;\mathrm{matrix}\;\mathrm{of}\;n\;\times \;18{\left[\begin{array}{c} a_{1}\left(1\right),a_{2}(1).....{\dot{a}}_{3}a_{3}(1)\\ .\\ .\\ .\\ .\\ a_{1}(n),a_{2}(n).....{\dot{a}}_{3}a_{3}(n) \end{array}\right]}^{T},$$

Thus, the order coefficient C becomes the vector of 18 × 1, but the number of equations n significantly exceeds 18. Simple algebraic methods cannot solve such systems, requiring the use of specialized techniques. The least squares method is commonly used to address this by minimizing the difference between the predicted and observed values. Further specific methods to solve this, discussed later, include iterative techniques and regularization to improve the accuracy of the model.

### 2.2. Improved Model

When applying the traditional T-L model for magnetic compensation, the required cosine value is generally obtained via a three-axial magnetic fluxgate magnetometer. However, this method exhibits significant limitations due to its inherent poor precision, high susceptibility to interference noise, and ease of disruption by external magnetic fields. As a result, the accuracy of the cosine values derived from the fluxgate magnetometer during flight operations is compromised. Consequently, it is recommended to avoid depending solely on the three-axis fluxgate magnetometer for acquiring direction cosine values [27]. Therefore, this article provides an alternative approach that leverages the inertial navigation system and the GPS to supplement the three-axial fluxgate sensor, and the cosine value required for the compensation matrix is obtained by obtaining the gesture angle.

This paper first introduces the definitions of the geographic coordinate system and the aircraft coordinate system.

The geographical coordinate system is based on an abstract model of Earth, specifically an ellipsoid. This ellipsoid is rotated around its axis, with the north end designated as the Arctic and the south end as the South Pole. In this context, the aircraft carrier is visible as a point that is close to the surface, with its X axis pointing north, the Y axis pointing south, and the Z axis perpendicular to the ground, pointing to the center of Earth.

**Figure 2:**
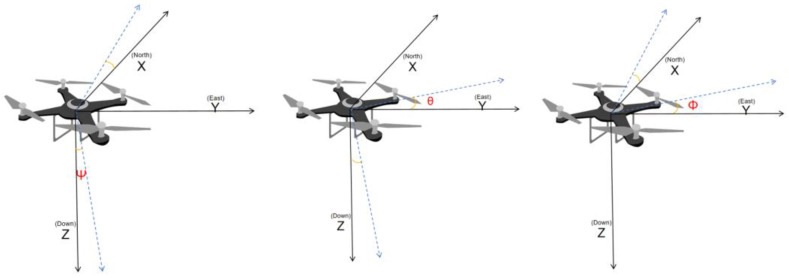
Geo-air coordinate variations.

The aircraft coordinate system is the origin O of the aircraft center, where the X axis is parallel to the fuselage, pointing in the direction of the machine, the Y axis is perpendicular to the fuselage, pointing in the direction of the wing, the Z axis perpendicular to the machine’s nose, pointing to the ground, and following the left-hand rule. As shown in Figure 2, because UAVs maneuver in the air, the coordinate system experiences changes in orientation, resulting in various attitude angles. The rotation of the aircraft coordinates defined around the X axis is the angle of roll, denoted as θ. The rotation of the aircraft coordinates defined around the Y axis is the angle of pitch, called Ψ. The rotation of the aircraft coordinates defined around the Z axis is the angle of yaw, called Φ. These three angles are flying gestures, which can be used for the transformation of the geographic coordinates and the aircraft coordinates.

The geographical coordinate and aircraft coordinate transformation matrices are as follows:

(15) $$W_{r}=\left[\begin{array}{ccc} 1 & 0 & 0\\ 0 & cos\;\theta & sin\;\theta \\ 0 & -sin\;\theta & cos\;\theta \end{array}\right]$$

This is the coordinate change matrix of roll action.

(16) $$W_{p}=\left[\begin{array}{ccc} cos\;\Psi & 0 & -sin\;\Psi \\ 0 & 1 & 0\\ sin\;\Psi & 0 & cos\;\Psi \end{array}\right]$$

This is the coordinate change matrix of pitch action.

(17) $$W_{y}=\left[\begin{array}{ccc} cos\;\Phi & sin\;\Phi & 0\\ -sin\;\Phi & cos\;\Phi & 0\\ 0 & 0 & 1 \end{array}\right]$$

This is the coordinate change matrix of yaw action.

Multiplied by the rolling, pitching, and yaw matrices, it is called the coordinate change matrix.

(18) $$\varphi =\left[\begin{array}{ccc} 1 & 0 & 0\\ 0 & cos\;\theta & sin\;\theta \\ 0 & -sin\;\theta & cos\;\theta \end{array}\right]\left[\begin{array}{ccc} cos\;\Psi & 0 & -sin\;\Psi \\ 0 & 1 & 0\\ sin\;\Psi & 0 & cos\;\Psi \end{array}\right]\left[\begin{array}{ccc} cos\;\Phi & sin\;\Phi & 0\\ -sin\;\Phi & cos\;\Phi & 0\\ 0 & 0 & 1 \end{array}\right]$$

The whole equation of the coordinate change can be written as

(19) $$[H_{X},H_{Y},H_{Z}]=\varphi \times [H_{x},H_{y},H_{z}]$$

where ${\;H}_{X}$, ${\;H}_{Y}$, and ${\;H}_{Z}$ are the components of the magnetic field on the aircraft axis,${\;and\;H}_{x}$, ${\;H}_{y}$, and ${\;H}_{z}$ are the components of the magnetic field on the three axis of the geographic coordinate system. From this equation, it is evident that if the components of the initial magnetic field in the geographic coordinate system are known, along with the real-time attitude angles obtained from the inertial navigation system and the GPS in the motion of the aircraft, the three-axis magnetic component of the real-time aircraft coordinate system can be determined. Using the geographic coordinate system, the original geomagnetic field can only be obtained [28] in the international geomagnetic reference field (IGRF). The international geomagnetic reference field model (IGRF) is a very important magnetic query tool. With this model, you can obtain the total strength F, horizontal strength H, and horizontal strength Z. X and Y are the northern and eastern components of H, and the magnetic angle D and the magnetic dip I can be calculated ($H_{x}$, ${\;H}_{y}$, ${\;H}_{z})$ by using these data.

By means of the method of coordinate variation above, we can obtain a method that does not require a fluxgate sensor and obtain the triaxial magnetic component. By combining the geo-air coordinate transformation, we can obtain a new T-L equation, as follows:

(20) $$H_{g}=H_{x}+H_{v}+H_{b}=\sum_{i=1}^{3}x_{i}e_{i}+H_{e}\sum_{i=1}^{3}\sum_{j=1}^{3}v_{ij}e_{i}e_{j}+H_{e}\sum_{i=1}^{3}\sum_{j=1}^{3}b_{ij}{\dot{e}}_{i}e_{j}\;i,j=1,2,3$$

(21) $$H_{g}=J\times F$$

Here, $\;H_{g}$ is still the UAV interference field, but now *J* represents a vector, including [$j_{1}$, $j_{2}$, $j_{3}$, $j_{1}j_{1}$, $j_{1}j_{2}$, $j_{1}j_{3}$, $j_{2}j_{2}$, $j_{2}j_{3}$, $j_{3}j_{3}$, $j_{1}j_{1}$, $j_{1}j_{2}$, $j_{1}j_{3}$, $j_{1}j_{3}$, $j_{2}j_{1}$, $j_{2}j_{2}$, $j_{2}j_{3}$, $j_{3}j_{1}$, $j_{3}j_{2}$, $j_{3}j_{3}$]. $j_{i}$ represents the derivative of the three direction cosines of time, and F represents the unknown solution coefficient of 18, *F* = [f1, f2, f3, …, f18].

Here, $e_{i}$ represents the direction cosines of the equation, cos α, cos β, and cos γ, ${\dot{e}}_{i}$ represents the derivative of the three direction cosines of time, $x_{i}$ represents the coefficient of the permanent field, $v_{ij}$ represents the coefficient of the induction field, and $b_{ij}$ represents the coefficient of the eddy current field. The direction cosine $s\;e_{1}$, $e_{2}$, and $e_{3}$ are derived from the following formula:

(22) $$[e_{1},e_{2},e_{3}]=\varphi \times [c_{1},c_{2},c_{3}]$$

(23) $$c_{1}=\frac{H_{x}}{\sqrt{H_{x}^{2}+H_{y}^{2}+H_{z}^{2}}}$$

(24) $$c_{2}=\frac{H_{y}}{\sqrt{H_{x}^{2}+H_{y}^{2}+H_{z}^{2}}}$$

(25) $$c_{3}=\frac{H_{z}}{\sqrt{H_{x}^{2}+H_{y}^{2}+H_{z}^{2}}}$$

where $H_{x}$, ${\;H}_{y}$, and $H_{z}$ are acquired from IGRF.

The modified model recommended in this article combines the traditional T-L Equation (13) and the new T-L Equation (20), which is processed after the coordinate changes and can be written as:

(26) $$H=H_{d}+H_{g}=A\times C+J\times F=M\times N$$

When the data point reaches n, M is the vector of n×36, and N is the unknown vector of 36 × 1.

**Figure 3:**
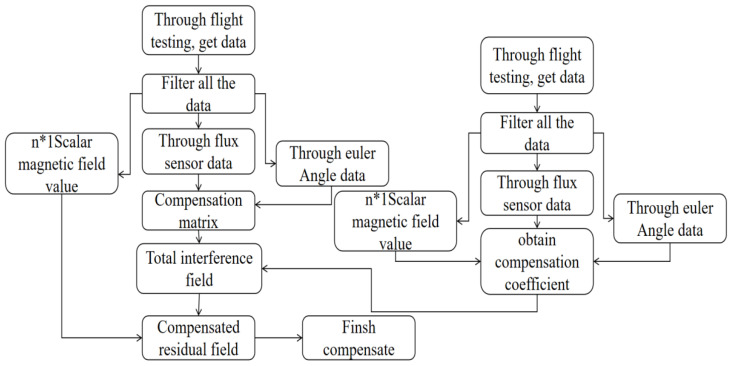
Overall aeromagnetic compensation flow chart.

Figure 3 shows the overall flow chart of aeromagnetic compensation. The steps are as follows.

(1) Data were obtained by the optical pump magnetometer, the three-axial magnetic fluxgate magnetometer, the inertial navigation instrument, and the GPS.
(2) All the collected data were filtered to eliminate the influence of the geomagnetic gradient.
(3) The data from the three-axis fluxgate magnetometer, inertial navigation instrument, and GPS were used to calculate the new direction cosine matrix.
(4) The equation was solved with the data from the optical pump magnetometer and the previously obtained direction cosines to derive 36 compensation factors.
(5) New data were obtained through the new flight circle.
(6) Steps (2) and (3) were repeated with these new data.
(7) The total interference magnetic field was obtained by multiplying the compensation matrix with the compensation coefficients obtained previously.
(8) All magnetic field data were compensated by subtracting the interference magnetic field.

In the course of aeromagnetic compensation, we use the standard deviation (std) to evaluate the data before and after compensation. The ratio of the standard deviation of the compensation is defined as IR, which is used to evaluate whole compensation process, as follows:

(27) $$IR=\frac{STD\left(H_{b}\right)}{STD\left(H_{a}\right)}$$

$H_{b}\;and\;H_{a}$ represent the data before and after compensation, respectively.

### 2.3. Proposed Optimization Algorithm

Because the equation derived above is overdetermined, there is no accurate solution, and one must resort to optimization algorithms to approximate the solution. Common methods include LS, SVD, RR, etc. However, because the matrix has multicollinearity problems, these methods can yield inaccurate results. Each one has inherent shortcomings, leading to errors in the final coefficients. Therefore, this article recommends a novel optimization algorithm, combining the principles of the Newton iteration, which we call Lasso regularization Newton iteration, or LRNM. The amount of data obtained during the course of magnetic compensation is extensive, leading to multiple common linear problems and causing model instability, known as “pathological equations”. Lasso regression addresses this by adding a regularization term (L1 norm penalty) to the loss function, effectively shrinking some coefficients to zero. This not only helps prevent overfitting but also facilitates feature selection, simplifying the model and enhancing its generalization capability [29]. Additionally, the regularization properties of Lasso regression allow it to handle complex datasets, even with high correlations among variables, while maintaining stability and interpretability. The Lasso regression target function is as follows:

(28) min||y−xβ||^2+λ||β||1

||y−xβ|| is the sum of the residual sum of the sum of squares, the error of measuring the prediction value and the real value. ${\lambda ||\beta ||}_{1}$ is the sum of the absolute value of all the elements in the β vector, which is the L1 regularization term. λ is the regularization parameter, and the strength of the regularization term is controlled.

Lasso return is insensitive to noise and abnormal values because it is used in L1 regulation. The basic idea of the Newton iteration method is to use the gradient information and the second derivative of the current iteration point to create a quadratic approximation of the target function, and then identify the minimum point of the quadratic function as the new iteration point. This process will repeat until the minimum point of the function is found. Mathematically, this can be achieved by solving the first derivative of the function and setting it to zero to find the resting point.

**Figure 4:**
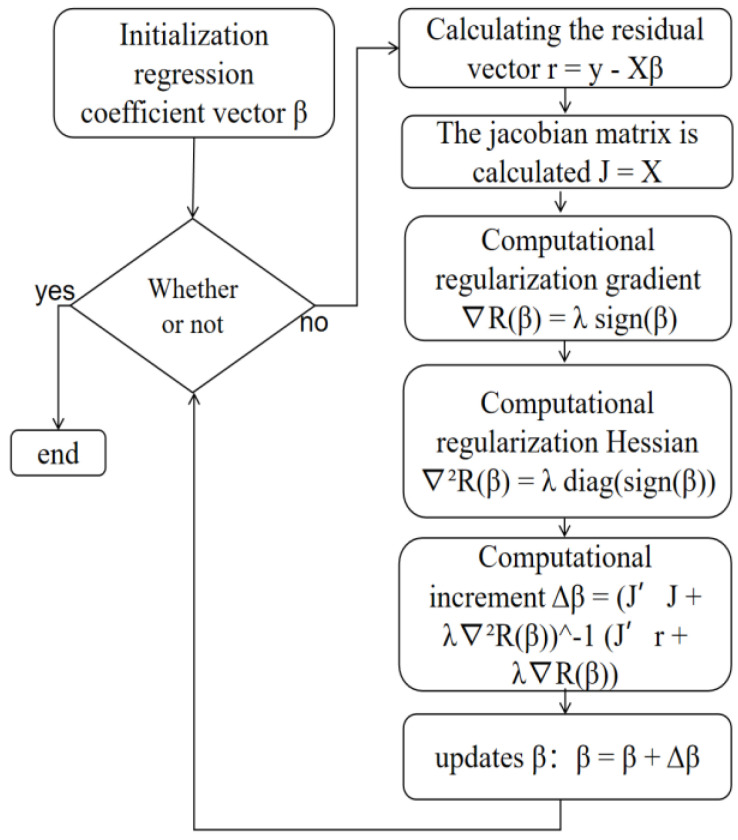
Flow chart of LRNM.

The method recommended in this article integrates both Lasso regression and Newton iteration to solve the original equation, thereby reducing the multicollinearity to some extent, as illustrated Figure 4:

X is the input matrix, y is the target vector, the λ is the regularization parameter. The Newton iteration method, with its ability for data processing, rapid convergence, and noise resistance, combined with the Lasso regularization, which prevents the optimization algorithm from getting stuck in local minima, results in a more stable algorithm. This integrated approach can better handle pathological equations and reduce the problem of multicollinearity to a certain extent. Compared to traditional methods, such as singular value decomposition (SVD), least squares (LS) have good progress. In this paper, the traditional method of solving is compared with the new method. LRNM shows that it has good precision in the compensation coefficient solution and excellent performance in the magnetic compensation effect.

## 3. Experiments and Results

In order to verify the model and solution proposed in this paper, experimental verification was carried out through flight experiments in the surrounding waters of Hainan Province, South China. The aircraft was equipped with an optical pump magnetometer, a three-axial magnetic fluxgate magnetometer, an inertial navigation instrument, and a global positioning system (GPS). The optical pump magnetometer provided the total magnetic field data during the flight. The three-axis magnetic fluxgate magnetometer provided the magnetic field data for the X, Y, and Z axes under the aircraft coordinate system. The inertial navigation instrument provided the attitude angles of the aircraft during flight, including the pitch angle, roll angle, yaw angle, flight height, and flight speed. The GPS provided the real-time longitude and latitude of the flight. The sampling rate of the data was 10 Hz, and a band pass filter (0.05–0.6 Hz) was used to filter the data from the three-axis magnetic fluxgate and the optical pump magnetometer, reducing the influence of the geomagnetic gradient. Two sets of compensation flight experiments were carried out, referred to as A and B.

**Figure 5:**
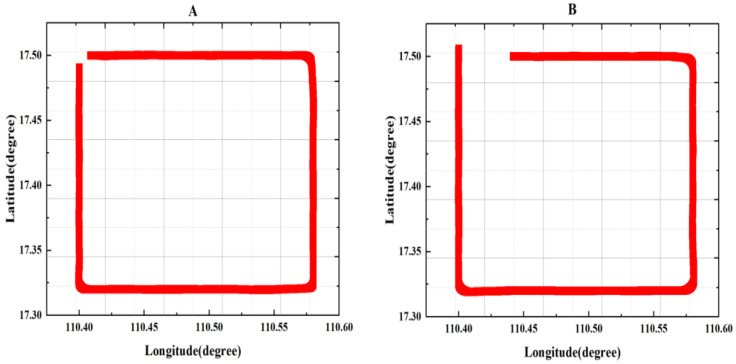
Flight compensation circle, including calibration circle (**A**) and test circle (**B**).

In Figure 5, two major compensation flight circles are depicted, both occurring nearly at the same location. The longitude path spans approximately 0.15 degrees, and the latitude span is also about 0.15 degrees. Both flight paths were counterclockwise. The flight pattern started from north to south, then went from west to east, followed by going from south to north, and finally going from east to west. During each phase, three sets of maneuvers—rolling, pitching, and yawing—were performed. Each maneuver was conducted in every direction, with the amplitude of motion slightly greater than described in previous studies [6,30]. We made roll angles of ±15°, pitch angles of ±6°, and yaw angles of ±10°. The purpose of this design was to reduce the multicollinearity problems in the compensation matrix to a certain extent, and it is better to compensate for different columns in the compensation matrix [25].

**Figure 6:**
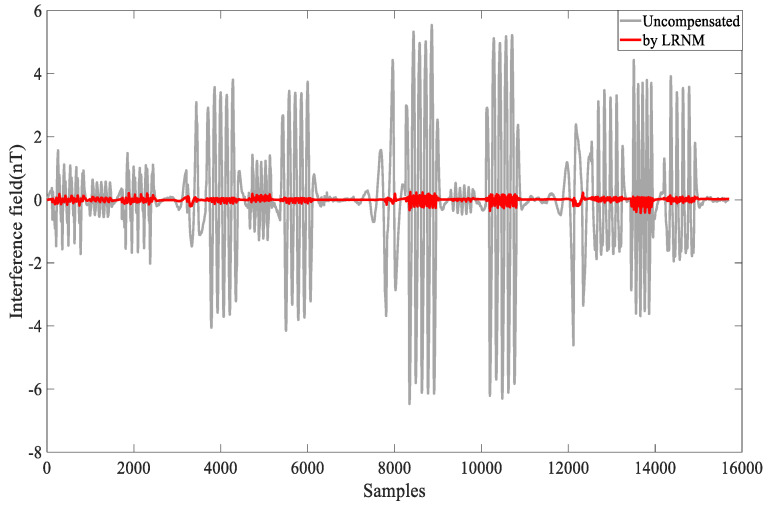
Data A, compensated by LRNM.

The overall flight altitude was near 3200 m. The aircraft was equipped with instruments and equipment and performed circular flights and specific maneuvers during the process. Flight circle A was designated as the compensation flight, while flight circle B served as the verification flight to assess the effect of the compensation coefficients. Data from flights A and B, which the optical pump magnetometer processed (through the band pass filter of 0.05–0.6 Hz), are shown in Figure 6.

**Figure 7:**
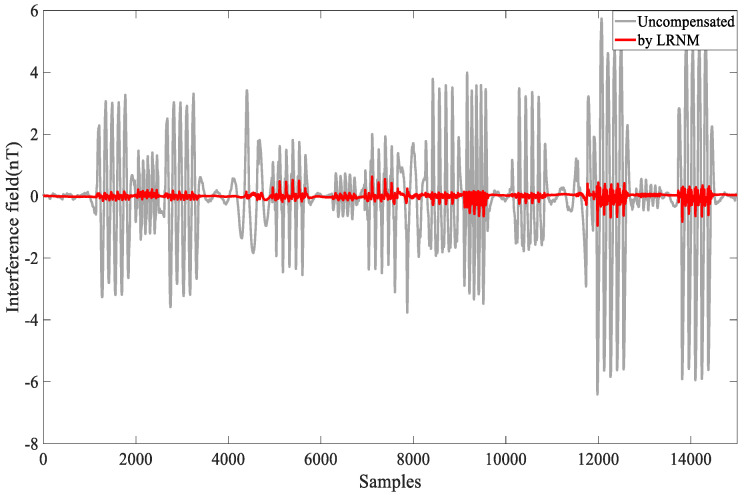
Data B, compensated by LRNM.

The compensation results are shown in Figure 7. The gray part represents the uncompensated interference field, while the red part represents the compensated field. This visual representation allows us to clearly observe the effects of the compensation, highlighting the reduction in interference achieved through the applied compensation techniques.

Comparing other proposed methods, the least squares method (LS) and singular value decomposition method (SVD), the difference in their magnetic compensation data applied in the large interference field was not significant. This comparison indicates that while all methods provided a degree of improvement, the performance variations between LS and SVD in high-interference environments are minimal.

As shown in Figure 7, flat flight C data were obtained without any maneuvers during this period and used to verify the effective of the compensation coefficients. The compensation coefficient was still solved by the data from flight circle A. Then, the coefficient was used to compensate for the flat flight C, validating the algorithm’s capability.

## 4. Results Analysis

In Figure 5, for calibration circle A, the std = 2.211 nT is slightly larger than the values mentioned in many other papers. The reasons for this are as follows: The flight in this article involved nearly 16,000 sample points. During the flight, we experienced disturbances from the gradient of Earth’s magnetic field. To reduce the multicollinearity problems to some extent, we selected larger compensation maneuvers, which introduced additional magnetic interference during the compensation process. Additionally, because the aircraft platform was large and carried many instruments, compensating for the large interference field was challenging. Despite these challenges, the combined compensation model and the optimized algorithm adopted in this paper demonstrated effective results, as shown in Figure 6 and Figure 7, and Table 1 and Table 2. Flight circle A was compensated for by a compensation factor it obtained, IR = 20.46, and the same compensation coefficient was used for compensation in circle B, IR = 17.67.

**Figure 8:**
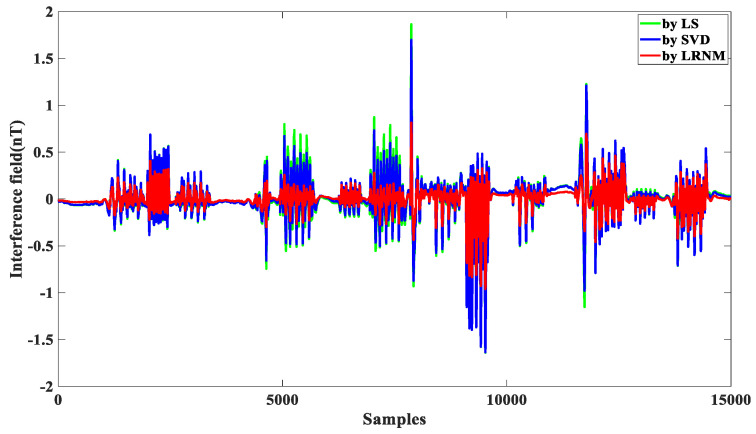
Comparison of three methods. Green represents LS, blue represents SVD, and red represents LRNM.

In order to further demonstrate the effectiveness of LRNM in this article, as shown in Figure 8, we compared the traditional methods, like LS and SVD, and we saw that the compensation effect of LRNM was advantageous. As illustrated in Table 3, compared to the SVD method, IR = 13.89, an improvement of 27%; compared to the LM method, IR = 13.18, an improvement of 34%. The effectiveness of the LRNM optimization algorithm is obvious.

**Figure 9:**
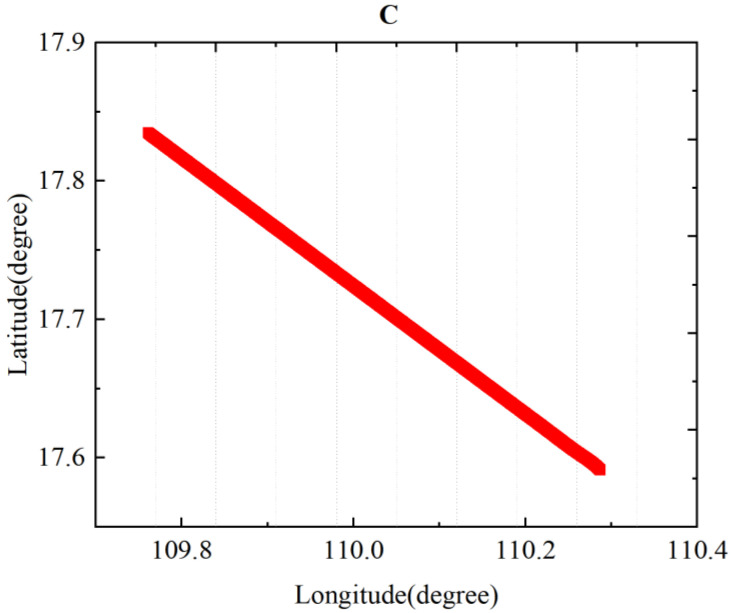
Flat flight data.

**Figure 10:**
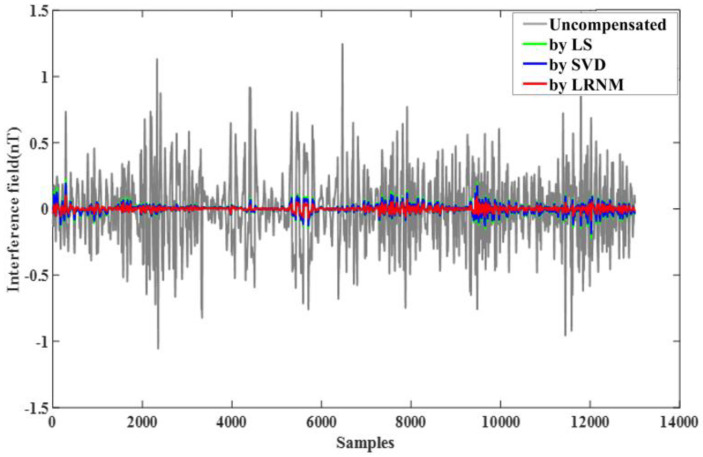
The flat flight data compared to three methods. Green is LS, blue is SVD, and red is LRNM.

Finally, in order to test the UAV flight experiment, the model and the optimization algorithm were further evaluated using flat flight data from flight circle C, as shown in Figure 9. During this period, no maneuvers were carried out, and all interference noise was generated by the large aircraft platform and the various instruments and equipment on it, resulting in an std of 0.249 nT. This reflects the high-interference field caused by the UAV and its equipment, as shown in Figure 10. However, by using the compensation scheme proposed in this paper, the compensated std was reduced to 0.0158 nT, achieving an IR of 15.73, which demonstrates very good performance. Compared to the SVD method (IR = 12.15), the improvement ratio increased by 29%, and compared to the LS method (IR = 11.64), the improvement ratio increased by 35%. The effectiveness of the LRNM optimization algorithm is evident.

## 5. Conclusions

Magnetic compensation directly affects magnetic detection, and this paper presents a series of compensation strategies for more practical large multi-load UAVS. First, by addressing the limitations of the traditional model, where the triaxial magnetic fluxgate magnetometer exhibits poor anti-magnetic interference capability, we used data from the three-axis magnetic fluxgate, inertial navigation system, and GPS to obtain direction cosine information collectively. The initial compensation matrix obtained from the three-axis fluxgate was expanded into a compensation matrix with 36 coefficients. Second, to mitigate multicollinearity issues, this paper implemented larger maneuvers during the flight compensation, which helped reduce the multicollinearity to a certain extent. Finally, to handle the large interference fields resulting from the combination of the large UAV platform and substantial compensation actions, we proposed a new optimization algorithm, the Lasso regularization Newton iteration (LRNM). This algorithm further reduced the multicollinearity problems. A comparison with the traditional LS and SVD methods showed that, for the verification circle flight B, the LRNM method increased the IR by 34% and 27% compared to LS and SVD, respectively. For flat flight lines, the LRNM method increased the IR by 35% and 29% compared to the LS and SVD, respectively. To sum up, this series of strategy designs can be combined to provide excellent compensation for large multi-load drones, which is crucial for practical aeromagnetic exploration.

In the future, aeromagnetic exploration will likely involve a large-scale UAV as a carrier with multiple sensors, so that a variety of tasks can be carried out. This type of model is required to have more effective aeromagnetic compensation. The current UAV manufacturing is increasingly utilizing composite materials, so the interference magnetic field generated by the fuselage will diminish. However, the presence of numerous electromechanical devices will generate more interference magnetic fields. Therefore, the future direction of aeromagnetic compensation may shift toward addressing interference from internal electromechanical equipment, which is a factor that needs careful consideration. Additionally, as the complexity of UAVs and their onboard systems grows, developing advanced algorithms and compensation strategies to handle these new sources of interference will become increasingly important. In conclusion, aeromagnetic exploration is poised to become an indispensable tool in many fields, making effective aeromagnetic compensation critically important. Ensuring precise and reliable magnetic data will be crucial for the success of these future UAV-based exploration missions.

## Figures and Tables

**Table 1:** The compensation coefficients of circle A were compensated for in A and B, and their IRs were determined.

Flight	Uncompensated	Compensate	IR
A	2.211 (nT)	0.108 (nT)	20.46
B	2.139 (nT)	0.121 (nT)	17.67

**Table 2:** The compensation coefficients obtained from circle A were compensated for in circle B using three optimization algorithms: LS, SVD, and LRNM.

Method	Uncompensated	Compensated	IR
LS	2.139 (nT)	0.161 (nT)	13.18
SVD	2.139 (nT)	0.154 (nT)	13.89
LRNM	2.139 (nT)	0.121 (nT)	17.67

**Table 3:** The flat flight data were compared to three methods, after being compensated for by the coefficients from calibration circle A.

Method	Uncompensated	Compensated	IR
LS	0.249 (nT)	0.0214 (nT)	11.64
SVD	0.249 (nT)	0.0205 (nT)	12.15
LRNM	0.249 (nT)	0.0158 (nT)	15.73

## Data Availability

The data presented in this study are available on request from the corresponding author due to the acquisition of data is confidential information by design in a very special way.
